# Metformin Induced AMPK Activation, G0/G1 Phase Cell Cycle Arrest and the Inhibition of Growth of Esophageal Squamous Cell Carcinomas *In Vitro* and *In Vivo*

**DOI:** 10.1371/journal.pone.0133349

**Published:** 2015-07-21

**Authors:** Xianbin Cai, Xi Hu, Xiaojun Tan, Weijie Cheng, Qinjia Wang, Xiaofeng Chen, Yinghong Guan, Chong Chen, Xubin Jing

**Affiliations:** 1 Department of Gastroenterology, The First Affiliated Hospital of Shantou University Medical College, Shantou, Guangdong 515031, China; 2 Department of Internal Medicine, Chancheng District Central Hospital, Foshan, Guangdong 528031, China; Harvard Medical School, UNITED STATES

## Abstract

Esophageal squamous cell carcinomas (ESCC) have become a severe threat to health and the current treatments for ESCC are frequently not effective. Recent epidemiological studies suggest that the anti-hyperglycemic agent metformin may reduce the risk of developing cancer, including ESCC, among diabetic patients. However, the antitumor effects of metformin on ESCC and the mechanisms underlying its cell cycle regulation remain elusive. The findings reported herein show that the anti-proliferative action of metformin on ESCC cell lines is partially mediated by AMPK. Moreover, we observed that metformin induced G0/G1 phase arrest accompanied by the up-regulation of p21^CIP1^ and p27^KIP1^. *In vivo* experiments further showed that metformin inhibited tumor growth in a ESCC xenograft model. Most importantly, the up-regulation of AMPK, p53, p21^CIP1^, p27^KIP1^ and the down-regulation of cyclinD1 are involved in the anti-tumor action of metformin *in vivo*. In conclusion, metformin inhibits the growth of ESCC cells both in cell cultures and in an animal model. AMPK, p53, p21^CIP1^, p27^KIP1^ and cyclinD1 are involved in the inhibition of tumor growth that is induced by metformin and cell cycle arrest in ESCC. These findings indicate that metformin has the potential for use in the treatment of ESCC.

## Introduction

Esophageal carcinoma is the sixth most common cause of cancer-related deaths in the world [1]. In developing countries, the major histological type of esophageal carcinoma is ESCC. In general, in most ESCC cases, the disease is at an advanced stage at the time of its diagnosis. Even in the early stage of ESCC, 20% of patients experience a recurrence after curative esophagectomy [2]. Curative surgery, chemotherapy and radiotherapy have limited effects on ESCC due to various factors, including tolerance, the general condition of the patient, tumor stage and treatment cost. To date, despite the wide variety of ESCC treatment options that are available, the poor prognosis of ESCC remains a challenge for clinical medicine. Therefore, developing a more effective treatment of ESCC would be highly desirable.

Over the past 50 years, metformin has been widely used for the treatment of type 2 diabetes mellitus. Metformin improves insulin resistance and the metabolic syndrome, which are considered to be carcinogenic factors [3,4]. Recent epidemiological studies have shown that metformin reduces the risk of developing gastroenterological cancer, including ESCC in some diabetic patients [5]. Furthermore, it has been established that metformin exerts its anti-tumor effects on various cancerous cell lines *in vitro*, including breast cancer, prostatic cancer, colon cancer, and gastric cancer [6,7,8].

Prior studies have shown that metfromin exerts its anti-proliferative action partly by activating AMP-activated protein kinase (AMPK) [7,9]. AMPK is an important energy-sensing enzyme that maintains cellular energy homeostasis. It is activated by cellular stress which increases the adenosine monophosphate/ adenosine triphosphate (AMP/ATP) ratio, leading to the production of metabolic poisons, the development of hypoxia, glucose starvation, *etc*. [10]. In addition, it has been reported that metformin induces cell cycle restriction arrest by activating AMPK [6,9].

Cell cycle is a series of events leading to cell division and replication. Cell cycle progression can be restricted under conditions that are not suitable for DNA replication, including nutrient depletion, DNA damage and growth factor withdrawal [11,12]. Cyclin, cyclin-dependent kinases (CDKs) and CDK inhibitors (CDKIs) in the G1 phase interact with each other to regulate cell-cycle transitions and cell division. It has been shown that cyclin D1 and CDKs are essential for driving cells to pass the restriction point [13]. CDKIs, p21^CIP1^ and p27^KIP1^ can prevent inappropriate cyclin/CDK activity in the G1 phase [14,15]. Moreover, p53, a tumor suppressor and an up-stream regulator of p21^CIP1^, can indirectly affect the cell cycle [16]. These mechanisms associated with the restriction point control the order and timing of cell-cycle transition, whereas cell cycle regulatory functions are usually impaired in cancer cells. Hence, repairing cell cycle progression might be an effective strategy for the treatment of ESCC.

Although the effects of metformin on ESCC remain unclear. A recent study suggested the existence of an association between metformin and the inhibition of ESCC cell proliferation through the cell cycle, growth factor and miRNA regulation *in vitro* [17], but, as of this writing, no relevant *in vivo* evidence has been reported. In the present study, we report on an investigation of the anti-proliferative effect of metformin on ESCC cells in cultures, and on tumor growth in an ESCC xenograft animal model. Cell cycle regulatory proteins, as well as the role of AMPK were studied both *in vitro* and *in vivo*. The results indicate that metformin inhibits ESCC cell growth by causing cell cycle arrest and delaying tumorigenesis.

## Materials and Methods

### Cell line, cell culture and reagents

This study was approved and monitored by the Ethics Committees of the First Affiliated Hospital of Shantou Medical University. Human esophageal squamous cell carcinoma cell lines, EC109 [18,19] and EC9706 [20,21], were obtained from the Chinese Academy of Sciences and from The Cell Bank of Type Culture Collection of Chinese Academy of Sciences (CAS, Shanghai). Cells (1 × 10^5^) were grown in RPMI1640 medium (ICN; Biomedicals Inc.) supplemented with 10% fetal calf serum, 100 units/ml penicillin and 100 units/ml streptomycin (Invitrogen). 5-aminoimidazole-4-carboxamide-1-h-D-ribofuranoside (AICAR), 1,1-dimethylbiguanide hydrochloride (metformin) and Compound C (AMPK Inhibitor) were purchased from Sigma (St. Louis, MO).

### MTT assay

Cell proliferation was assessed by the (4,5-dimethylthiazol-2-yl)-2, diphenyl-tetrazoliumbromide (MTT) method. Cells were seeded at 3000 cells/well in 96-well plates containing the test compounds for the indicated times, and then incubated with 200 μL of a MTT solution (0.5 mg/ml in PBS) for 4h at 37°C. Optical density was determined using an enzyme-linked immunosorbent assay reader.

### Flow cytometry

For cell cycle analysis, cells were treated with or without AICAR or metformin for 24h. The cells (1 × 10^6^) were trypsinized and fixed in 70% ethanol overnight. The fixed cells were stained with propidium iodide (50 μg/ml) for 30 minutes at room temperature. The cells were detected by flow cytometry (Beckman Coulter Inc, Brea, CA), the number of cells 1 × 10^4^, excitation wavelength 488 nm and emission wavelength 630 nm. Cell cycle analysis was carried out using the WinMDI 2.9 software.

### Western blot analysis

ESCC tissues (30 mg) and cells (5× 10^5^) were sonicated in 300μl RIPA buffer supplemented with the protease and phosphatase inhibitors mixture (3μl), NaF (1mM 3μl), sodium orthovanadate (1mM 3μl), PMSF (1mM 3μl), homogenized, and then centrifuged (12000g for 5 minutes). The supernatant was used for protein determination using a BCA Protein Assay Kit (Pierce, IL, USA). Samples containing 30 μg of protein were added to SDS-PAGE loading buffer, heated for 3 minutes at 100°C and loaded on a gel. Electrophoretic transfer to polyvinylidene difuoride membrane was followed by immunoblotting with primary antibodies (Phoshpo-AMPKα (Thr-172), AMPKα, phosphor-ACC (Ser-79), ACC, CyclinD1, p53, p21^CIP1^ and p27^KIP1^, which were purchased from Cell Signaling Inc., and then with the secondary antibody rabbit horseradish peroxidase-conjugated anti-goat IgG (Vector Laboratories, Burlingame, CA, USA). Signals were developed by chemiluminescence using an enhanced chemiluminescence kit (Amersham, Piscataway, USA). The western blot images were acquired by a digital image processing system (Universal HoodⅡ76S/0608, Bio-Rad Inc, Hercules, CA). The gray value of each band in the imaging data was analyzed by quantity software (Quantity one, Bio-Rad Inc, Hercules, CA).

### Tumor xenograft model

Eight-week-old male athymic nude mice were purchased from Vital River Laboratories in Beijing. Nude mice were housed in a specific pathogen-free facility. Early passage EC109 cells were harvested and 1×10^6^ cells were implanted subcutaneously into each flank of each mouse. The animals were randomized into control and experimental groups (7–10 mice per group). In the prevention group, the treatment was initiated 7 days before implantation and continued to the end of the study; whereas in the treatment group the administration began two weeks after implantation and continued for the rest of the study. For intragastric administration, metformin was dissolved in physiological saline and administered once daily at a dose of 250mg/kg. The control group received isovolumic vehicle only. Tumor volume was measured with an external caliper every 2 days and was calculated as V = π/6(length × width × depth).

### Blood glucose analysis

After administration, water and food were withdrawn for 3h. Blood samples of mice were then collected by saphenous vein puncture, and glucose levels were measured by means of a glucose monitor (Omron, Beijing, China).

### Immunohistochemistry

Immunohistochemistry was performed on tumor tissues obtained from xenograft model mice. Slices were incubated in 3% (v/v) hydrogen peroxide for 10 minutes at room temperature. Antigen retrieval was done by heating tissue sections in 10mM (pH 6.0) citrate buffer for 20 minutes in a microwave oven. Tissue sections were incubated with primary antibodies (p53, and p21^CIP1^, which were purchased from Cell Signaling Inc) following the manufacturer’s recommendations. Sections were washed and incubated with secondary antibody (Vector Laboratories, Burlingame, CA, USA), and then with streptavidin—peroxidase solution. The color reaction involved the use of 3, 39-diaminobenzidine (DAB) with counterstaining by hematoxylin and eosin. The negative control involved omitting the primary antibody.

### Statistical analysis

Data were expressed as mean ± standard error (mean±SE). SPSS 17.0 was used for data variation analysis. The difference between two groups was calculated using the student t test. Results were considered to be statistically significant at *P* <0.05.

## Results

### Metformin inhibits cell proliferation in ESCC cells

**Fig 1:**
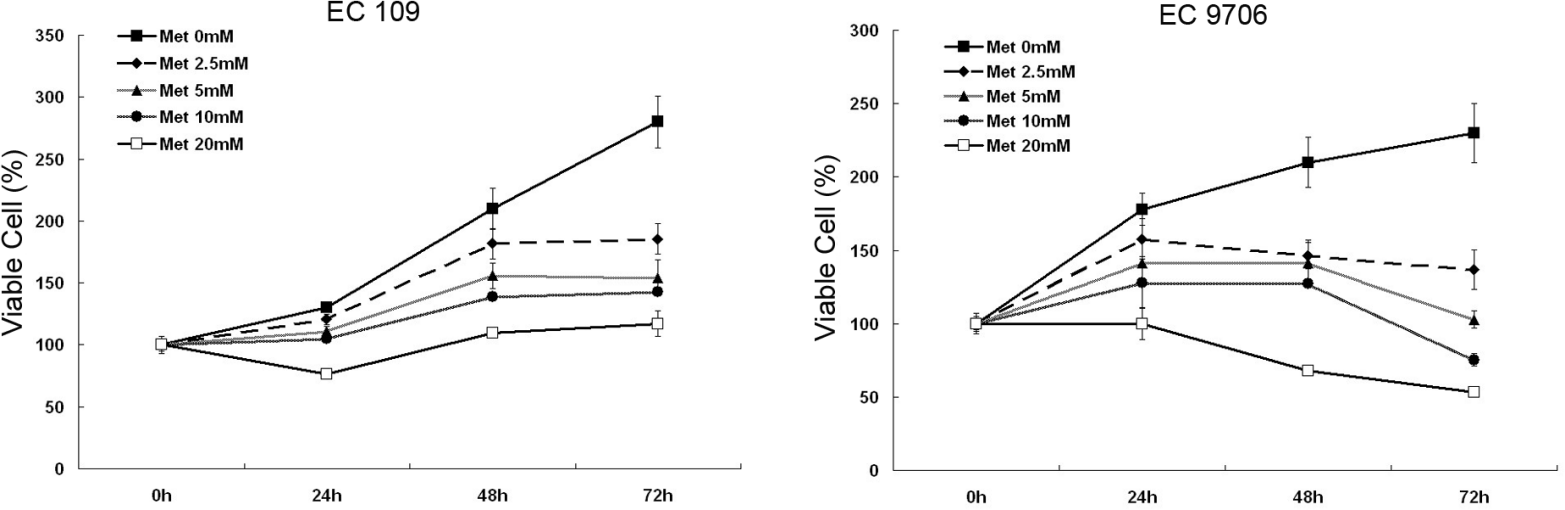
Metformin induced proliferation restriction in ESCC cells. EC109 and EC9706 cells were treated with 0, 2.5, 5.0, 10 or 20 mM metformin for 24h, 48h, 72h respectively. Cell viability was assessed by MTT. Data from three independent experiments are shown (Mean±SE).

To explore the role of metformin on the proliferation of ESCC cells, EC109 and EC9706 cells were treated with different concentrations of metformin for one to three days. As shown in Fig 1, cell viability was decreased with increasing concentrations of and the time of treatment with metformin. A dramatic suppression in the growth of the EC109 cell lines was observed after a 72-hour metformin (20mM) treatment. While in the EC9706 cell lines, a similar metformin treatment appeared to be more effective, resulting in a decline in the growth curve. These results show that metformin has an inhibitory effect on the growth of ESCC cells.

### Association between metformin-induced ESCC grwoth-inhibitory effect and AMPK activation

**Fig 2:**
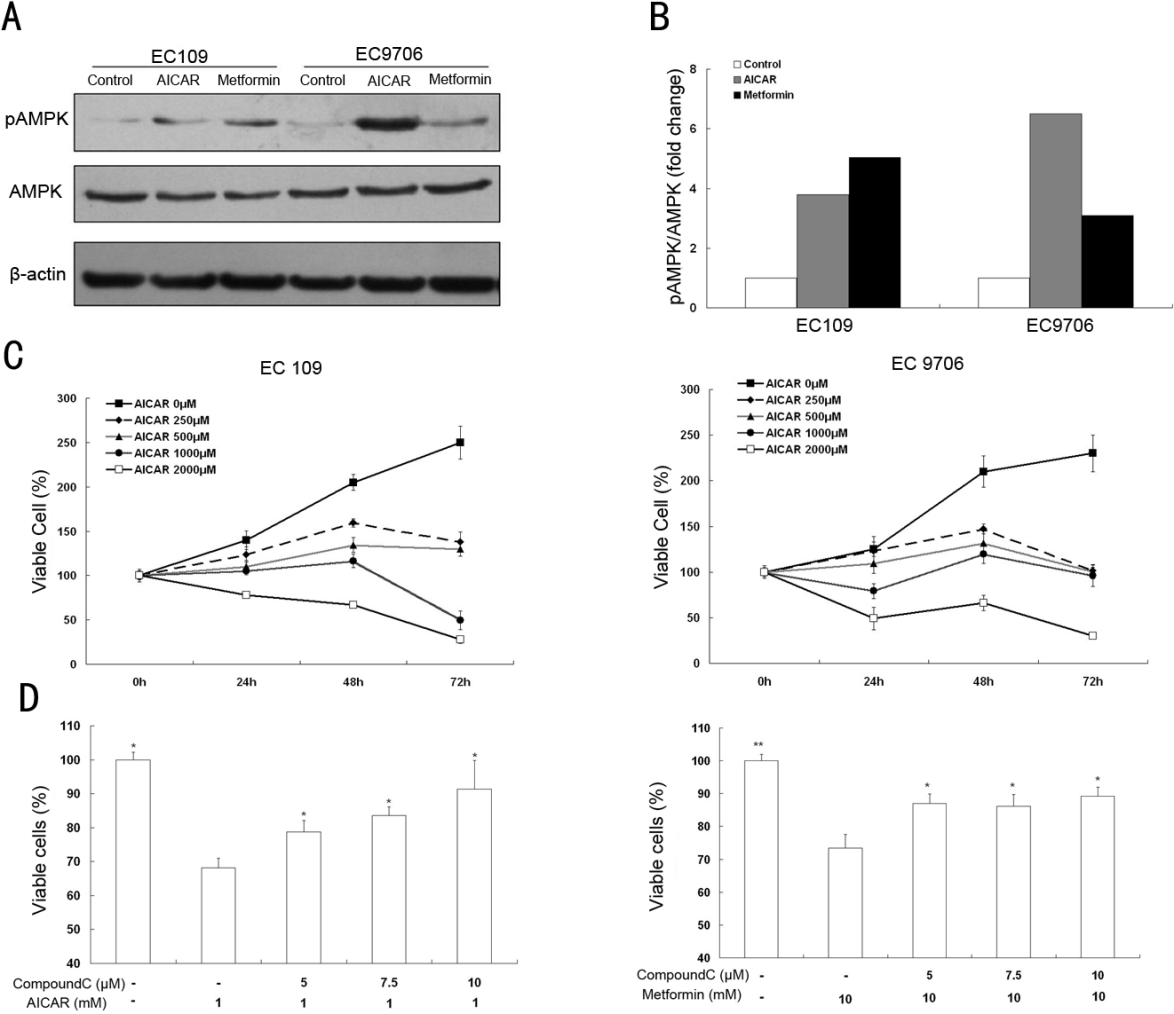
Association between metformin-induced ESCC grwoth-inhibitory effect and AMPK activation in vitro. (A) EC109 and EC9706 cells were treated with 1mM AICAR or 10mM Metformin for 24h. The level of phosphorylated AMPK in the ESCC cells was measured by Western blotting. (B) The histogram represents the ratio of phosphorylated AMPK to total AMPK which was normalized to the control group. Data is from three independent experiments. (C) The EC109 and EC9706 cells were treated with 0, 250, 500, 1000 or 2000 μM AICAR for 24h, 48h, 72h, respectively. Data from three independent experiments are shown (mean±SE). (D) The EC109 cells were pretreated with 0, 5, 7.5 or 10μM compound C for 30min, respectively, followed by a 24h metformin (10mM) or AICAR (1mM) treatment. Data represent menas±SE from three independent experiments. * *P*<0.05, ** *P*<0.01, comparing to the group treated with AICAR (or metformin) but not compound C.

Prior studies have suggested that metformin may exert its anti-tumor effects through the activation of AMPK (phosphorylated at Thr-172)[9,22], but this continues to be a subject of debate[23,24]. To determine whether metformin has the ability to activate AMPK, the AMPK phosphorylation levels in both ESCC cell lines were determined by western blot analysis. AICAR, an AMPK activator, was used here as a positive control. As shown in Fig 2A and 2B, the metformin and AICAR treatments increased the level of phosphorylation of AMPK in both ESCC cell lines compared to the untreated group. Further experiments were performed to verify the association between AMPK activation and cell growth inhibition in AICAR-treated ESCC cells. As expected, AICAR mimicked the metformin-induced ESCC growth-inhibitory effect in both dose- and time-dependent manners in both ESCC cell lines (Figs 1 and 2C).

To further explore the role of AMPK in the metformin-induced growth-inhibitory effect, compound C was used to selectively inhibit AMPK activation. EC109 cells were pretreated with compound C and then incubated with metformin (10mM) or AICAR (1mM) for 24h. Compared to the untreated groups, the compound C (10μM) treatment protected EC109 cells from AICAR and metformin induced growth inhibition (Fig 2D), suggesting that AMPK is directly involved in this process.

### Metformin induced G0/G1 phase cell cycle arrest in ESCC cells

**Fig 3:**
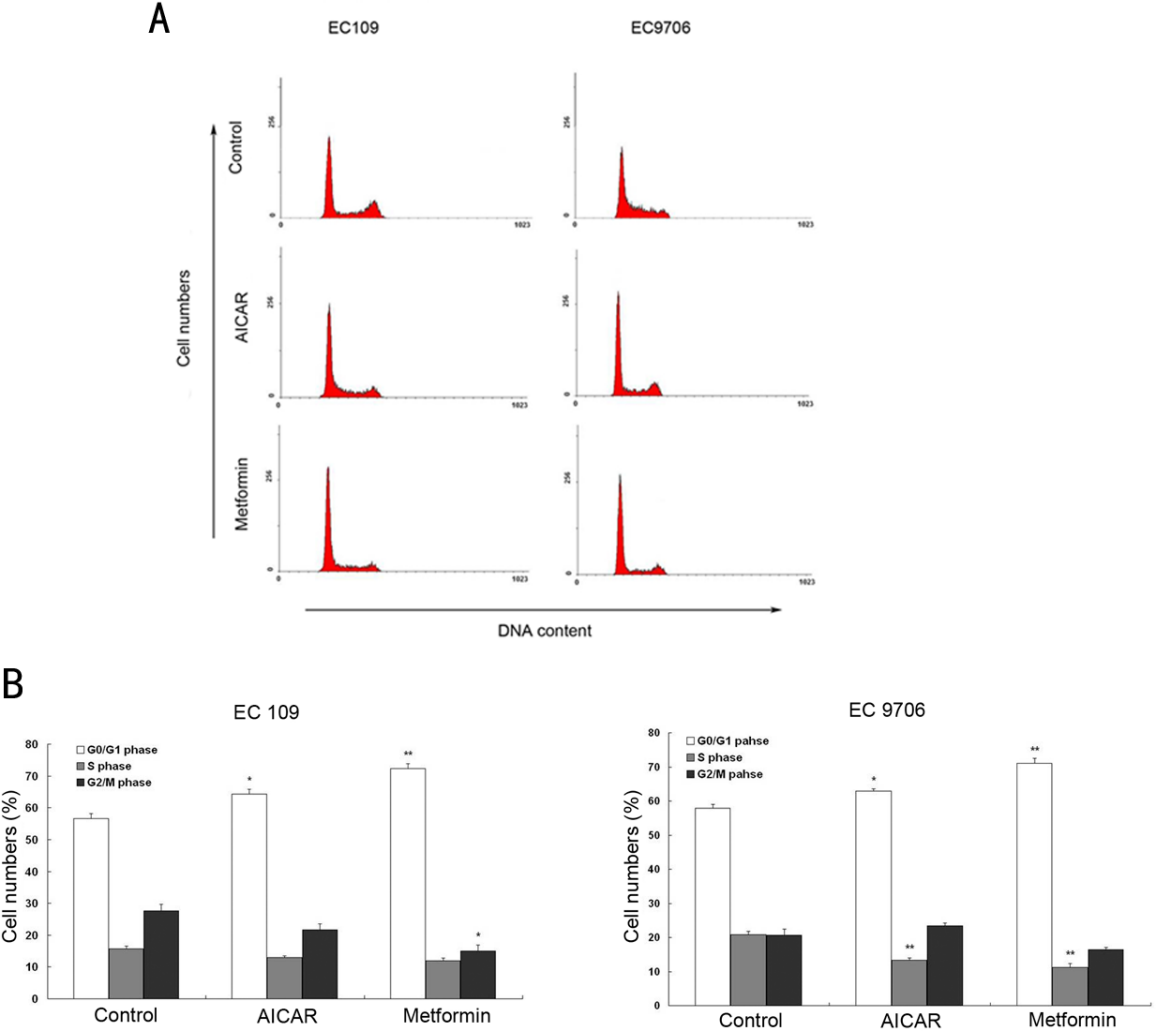
Metformin induced cell cycle arrest in ESCC cells. (A) Cells treated with 1mM AICAR or 10mM metformin for 24h were stained with propidium iodide, and analyzed by flow cytometry to estimate the amount of cells in each phase of the cell cycle. (B) The histogram represents the percentage of cells in each cell cycle phase. Data represent the means±SE from three independent experiments. * *P*<0.05, ** *P*<0.01, vs Control.

The unlimited cell division in cancer cells is mainly due to inappropriate cell cycle progression. To investigate the potential mechanisms responsible for metformin-induced cell growth inhibition, EC109 and EC9706 cells were treated with or without metformin (10mM) or AICAR (1mM) for 24h. Flow cytometry was performed on propidium iodide stained cells to determine the effects of metformin on the cell cycle progression. As shown in Fig 3A and 3B, G0/G1 cells had accumulated in both ESCC cell lines after the metformin and AICAR treatments. The number of S phase cells decreased significantly in the case of the treated EC9706 cells, whereas in the treated EC109 cells, a slight reduction in the number of G2/M phase cells was observed with no significant change in the number of S phase cells. The above data indicate that metformin arrests the cell cycle in the G0/G1 phase, thus inhibiting the proliferation of ESCC cells.

### Metformin up-regulated p21^CIP1^ and p27^KIP1^ in ESCC cells

**Fig 4:**
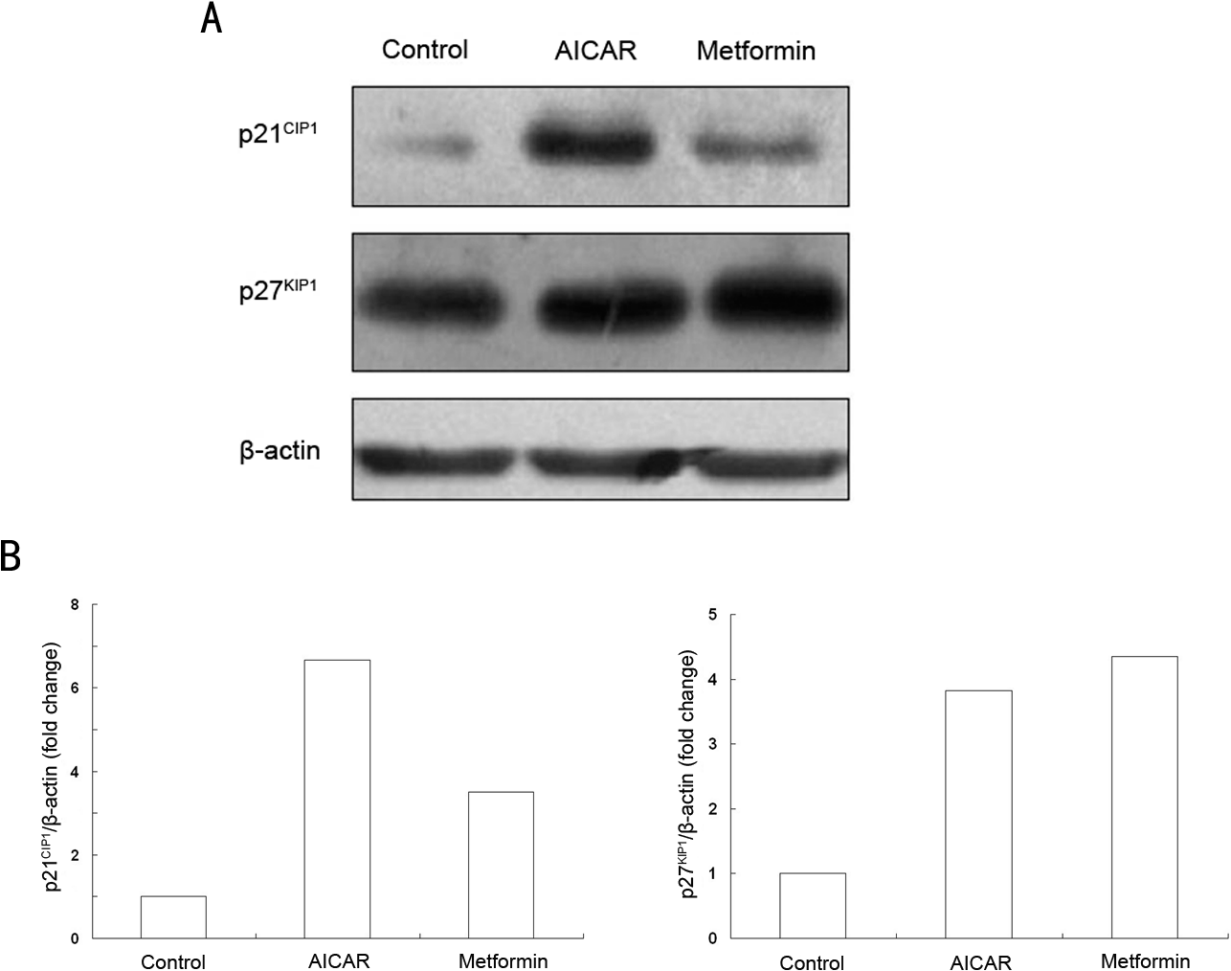
Metformin up-regulated p21CIP1 and p27KIP1 in ESCC cells. EC109 cells were treated with 1.0 mM AICAR or 10 mM metformin for 24h. (A) A representative Western blot of p21^CIP1^ and p27^KIP1^ in EC109 cells (B) The histogram represents the gray value ratio of the target protein to β-actin which was normalized to the control group. Data is from three independent experiments.

To investigate the molecular mechanisms responsible for metformin induced cell cycle arrest, western blot was used to examine the levels of main cell cycle regulatory proteins, p21^CIP1^ and p27^KIP1^, in EC109 cells. The findings clearly show that p27^KIP1^ and p21^CIP1^ were significantly up-regulated after a 24h treatment of 10mM metformin or 1mM AICAR (Fig 4).

**Fig 5:**
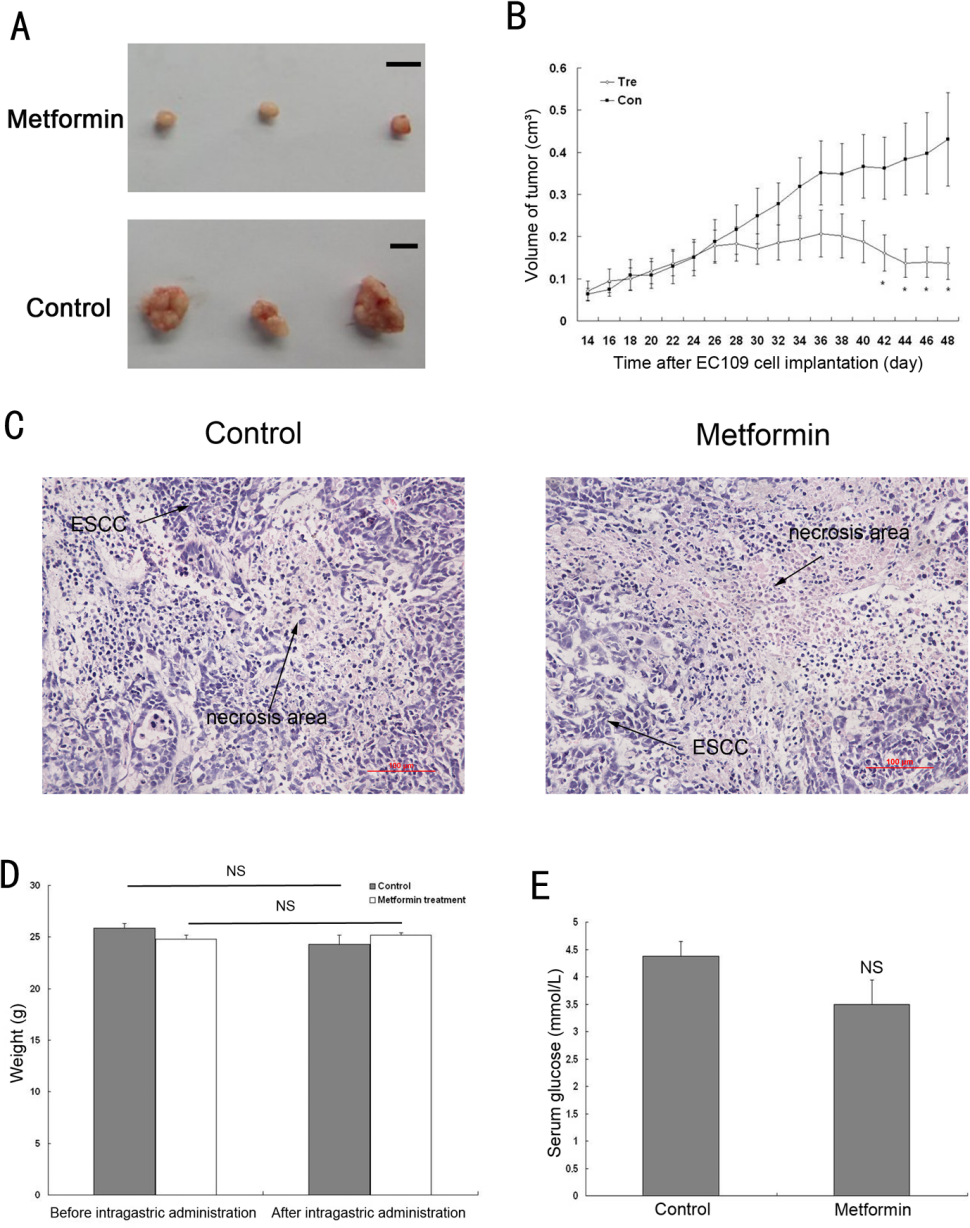
Metformin inhibited the growth of EC109 cell xenografts in nude mice. Xenografts were generated by the subcutaneous implantation of EC109 cells. Two weeks later, physiological saline (control) or metformin (250 mg/kg) were intragastrically administered and the feeding continued until the end of the study. Tumor volumes were measured every 2 days. (A) Photographs showing the representative xenograft tumors on metformin-treated or vehicle-treated nude mice (bar = 1centimetre). (B) Average tumor volumes of the EC109 cell xenografts. (C) Paraffin sections of excised tumors were assessed by H&E stain showing large necrosis area in both experiment and control groups. In necrotic area there were very few nuclei that were well stained by hematoxylin, and tissue was disintegrating and the morphologic details were not preserved (200× magnification; bar = 100 microns). (D) The body weight of all mice were measured the day before intragastric administration, and before euthanasia. Histograms show the average weight from the different groups. There was no difference before and after intragastric administration. (E) After intragastric administration, water and food were withdrawn.for 3h. The serum glucose was detected by blood glucose monitor. The serum glucose levels were not statistically different between the control and treated groups. Data represent mean ± SE. Tre: metformin treated group; Con: control group (n = 10; * *P*<0.05; ** *P*< 0.01; NS: no significance; vs. control).

The *in vitro* results indicate that metformin induces the inhibition of ESCC cell growth by blocking the cell cycle; we further examined the growth-inhibitory effect of metformin *in vivo*. Nude mice were administered with Metformin or physiological saline after the subcutaneous implantation of EC109 cells. The findings showed that metformin effectively suppressed the growth of the ESCC xenografts (Fig 5A and 5B). Tumors in the treatment group ceased growing 12 days after the metformin treatment (26 days after implantation). At 28 days later (42 days after implantation), the tumor size in the treatment group began to decline, leading to a significant difference compared with that in the control group. Interestingly, even though the tumor sizes were much smaller in the case of the metformin treatment, a pathological evaluation showed that these tumors had a similar scale of necrotic areas (Fig 5C).

Because the dosage of metformin (250 mg/kg/d) used in this *in vivo* study was much higher than that used in the treatment of diabetic patients (850 mg/d), the weight of the animals was measured to evaluate possible side effects caused by metformin. The metformin treatment had no significant effect on the body weight of the animals during the course of the treatment (Fig 5D). In addition, metformin caused a slight decrease in the fasting blood glucose levels in the nude mice, as shown in Fig 5E.

### The association between metformin and AMPK in vivo

**Fig 6:**
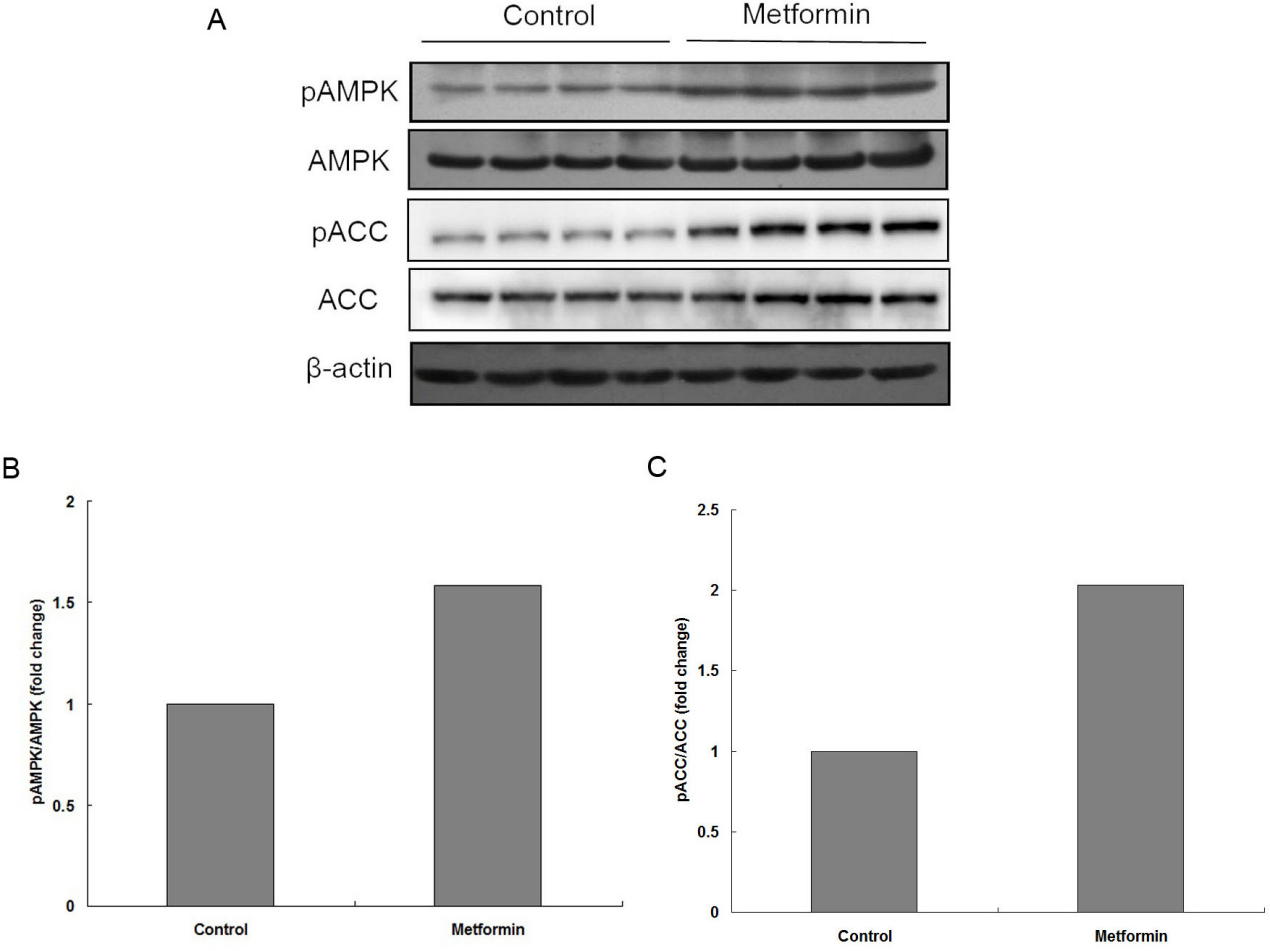
The association between metformin and AMPK in vivo. (A) Tissue extracts from 8 different samples of metformin-treated or vehicle-treated mice were immunoblotted with an antibody against phosphorylated AMPK, total AMPK, phosphorylated ACC and total ACC. (B and C) Diagrams show the gray value ratio of phosphorylated AMPK to the total AMPK and phosphorylated ACC to the total ACC which were normalized to the control group.

To detect whether metformin affects AMPK activity in EC109 cell xenografts, the levels of phosphorylated AMPK and its primary substrate phosphorylated Acetyl CoA carboxylase (ACC) [25] in tissue extracts were measured by western blot analysis. Consistent with the *in vitro* data, phosphorylated AMPK as well as phosphorylated ACC levels were up-regulated as the result of the systemic metformin treatment (Fig 6). The data confirmed that metformin induces the activation of AMPK, suggesting that AMPK might play an important role in metformin-induced growth inhibition.

### The effects of metformin on cell cycle regulators in vivo, including cyclinD1, p53, p21^CIP1^ and p27^KIP1^

**Fig 7:**
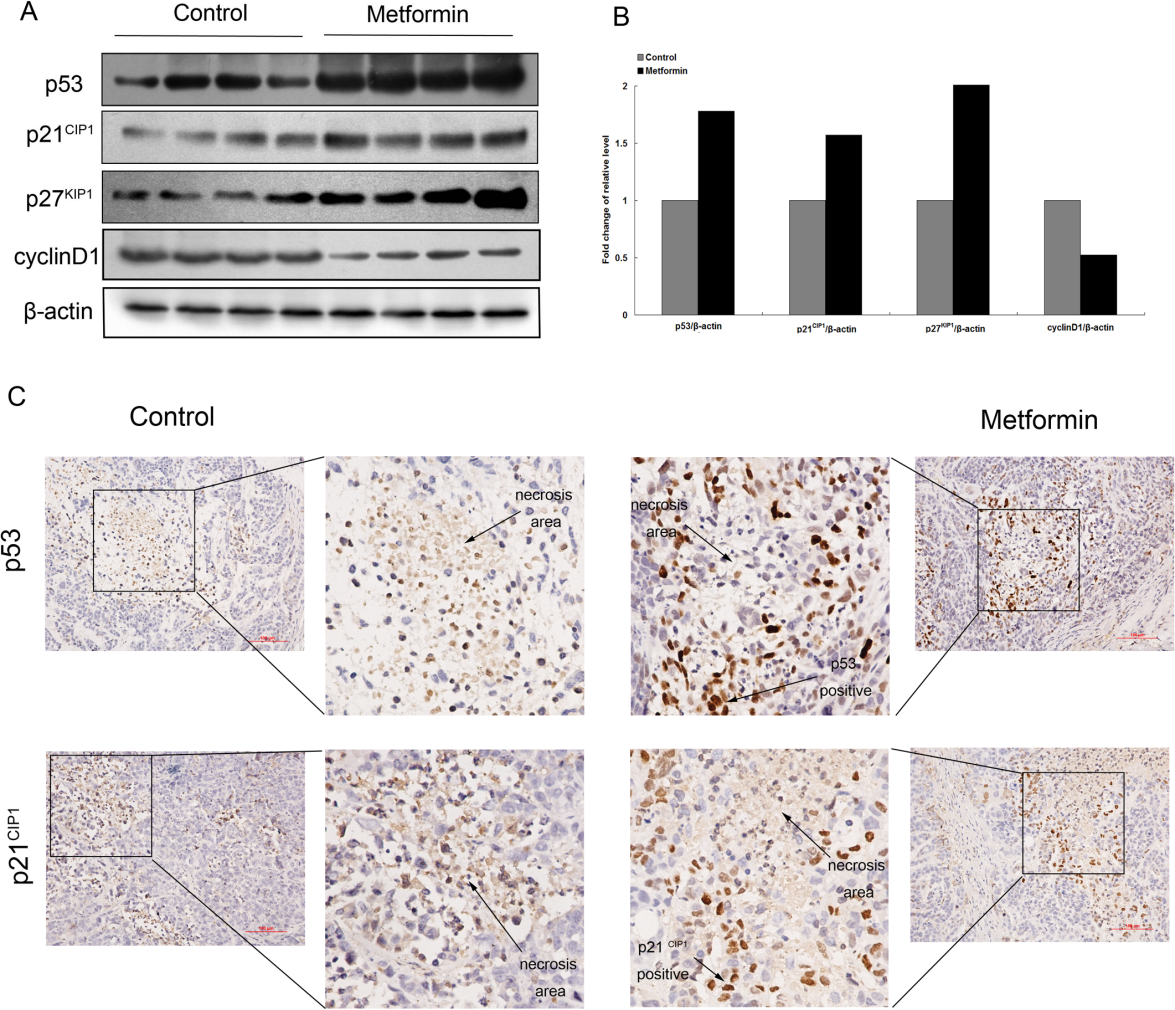
The effects of metformin on cell cycle regulators in vivo, including cyclinD1, p53, p21^CIP1^ and p27^KIP1^. (A) Tissue extracts from 8 different samples of metformin-treated or vehicle-treated mice were immunoblotted with antibodies against cyclinD1, p53, p21^CIP1^ and p27^KIP1^. β-actin was used as a loading control. Western blot analysis was performed. (B) Histograms represent the ratio of target protein to β-actin which was normalized to the control group. (C) Representative microscopic image of the immunohistochemiscal staining of p53 and p21 ^CIP1^ (200× magnification; bar = 100 microns).

To explore the molecular mechanisms responsible for these effects *in vivo*, we measured the levels of several cell cycle regulators by western blot or immunohistochemistry. It has been reported that p53, the up-stream regulator of p21^CIP1^, is required for AMPK-induced cell cycle arrest in mouse embryonic fibroblasts [11], and that cyclinD1 is essential for the G1/S transition in the cell cycle[13]. Therefore, the role of these two important cell cycle regulators were further examined *in vivo*. Similar to the results obtained in the *in vitro* experiments, the cyclin-dependent kinase inhibitors (CDKI) p21^CIP1^ and p27^KIP1^ were induced by metformin in ESCC xenograft models. As expected, p53 was strongly up-regulated after the metformin treatment, while the level of cyclinD1 was decreased (Fig 7A and 7B). Similarly, substantial numbers of p53 and p21^CIP1^ positive cells were observed around the necrosis area on the immunohistochmically stained tumor tissue sections from the metformin treatment. (Fig 7C).

### The association between metformin and ESCC prevention

**Fig 8:**
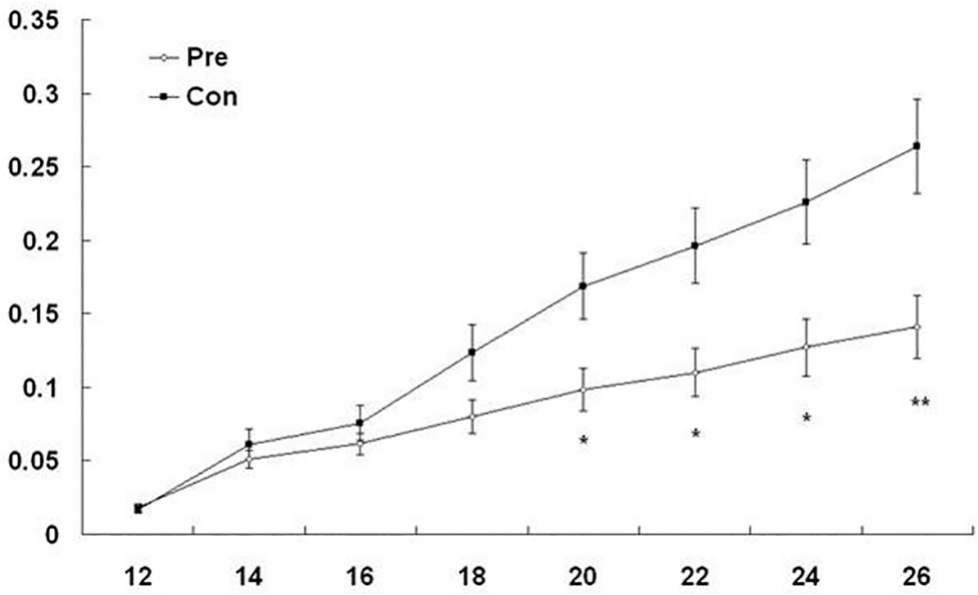
The effect of metformin for ESCC prevention. Animals were treated with metformin (250 mg/kg) or physiological saline (control) 7 days before tumor cell implantation. Tumor volumes were measured every 2 days. Graphs representing the average tumor volumes of EC109 cell xenografts. Data represent mean ± SE. Pre: metformin pretreatment; Con: control group (n = 12; * *P*<0.05; ** *P*< 0.01; vs. control).

To examine whether metformin could prevent ESCC, a metformin treatment was carried out 7 days before the implantation of tumor cells.Tumors were clearly visible at all implantation sites (12 out of 12) seven days after implantation in the untreated group, while, in the metformin pretreated group, only 67% sites (8 out of 12) had visible tumors. However, tumor incidence in the treated group increased to 100% at 14 days after implantation. The delay in the appearance of tumors confirms that tumor growth is inhibited by metformin. Furthermore, at 20 days after implantation, the average tumor size in the pretreatment group was significant smaller, only half the size of that in the control group (Fig 8). These data suggest that, even though prophylactic metformin treatments might not be able to prevent ESCC tumorigenesis, it can successfully decrease tumor size.

## Discussion

In the present study, we investigated the effect of metformin on ESCC growth *in vitro* and *in vivo*. A dose- and time-dependent proliferation restriction effect by metformin on two ESCC cell lines and a similar growth-inhibitory effect in a metformin-treated ESCC xenograft model was observed. The findings show that metformin induces G0/G1 phase cell cycle arrest through the down-regulation of cyclinD1 and the up-regulation of p53, p21^CIP1^ and p27^KIP1^ in ESCC. These results are consistent with findings reported in a previous study of the effects of metformin on cultured ESCC cell lines [17], and more importantly, the antiprolierative effect of metformin on ESCC is further validated in an animal model. To the best of our knowledge, this is the first study to show that metformin inhibits ESCC growth by inducing a cell cycle restriction point *in vivo*.

Metformin is an antidiabetic drug that activates AMPK, which plays an important role in maintaining cellular energy homeostasis. AMPK is activated under conditions of a shortage of energy [26], resulting in metabolic inhibition, which could lead to cell proliferation restriction. Additionally, AMPK can also cause cell cycle arrest, induce apoptosis, down-regulate growth factor receptor levels and inhibit angiogenesis [6,27,28,29]. AMPK activity is suppressed in tumors with oncogenic mutations, including Akt over activation and an LKB1 deficiency [26]. Hence, AMPK might be a molecular target for tumor treatment.

In this study, AMPK was found to be activated in response to a metformin treatment both *in vitro* and *in vivo* (Figs 1, 5, and 7). Furthermore, the *in vitro* data show that AICAR and a metformin treatment has similar effects on cell cycle regulation, implying AMPK might also be involved in the metformin-induced arrest in the cell cycle. Studies have indicated that metformin caused cell cycle arrest through AMPK and other regulators in various types of cancer [6,9]. It has been reported that AMPK activation is required in metformin-induced anti-tumor phenomena [9,22], while other studies showed that metformin exerts its action through AMPK-independent pathways [30] such as the mTOR, TGF-β pathway [23,31]. In our *in vitro* study, the AMPK inhibitor compound C compromised the restriction in proliferation induced by metformin in EC109 cells. However, the rescue of growth inhibition by high concentrations of compound C (10μM) in AICAR group (68.11% to 91.37%) appeared to be better than that in metformin group (73.36% to 89.23%), indicating that metformin may exert its anti-tumor action partly through the activation of AMPK in these cells (Fig 2D). However, the cellular and molecular mechanisms responsible for the action of metformin are complicated, in some cases they appear to extend beyond the AMPK activator. This explains, to some extent, in some carcinoma cells why metformin exerts its anti-tumor action via an AMPK-independent pathway.

The *in vivo* study further verifies that metformin suppresses tumor growth (Fig 5B), accompanied by the down-regulation of cyclin D1 and the up-regulation of p21^CIP1^, p27^KIP1^, and p53. Other animal experiments have shown that metformin prevents the development of tumorigenesis in the pancreas, lung and liver [24,32,33]. However, the preventive use of metformin in the ESCC xenograft model failed to prevent tumorigenesis, but significantly delayed tumor development (Fig 8). When metformin was given post-implantation, a significant difference in tumor size in the treatment group was not observed until 42 days (Fig 5B). In contrast, when metformin was administered prior to implantation, tumor size was significantly smaller in the pretreatment group at 20 days after implantation, which results in a much more manageable tumor.

Rapid cell proliferation and relatively weak angiogenesis in a tumor causes hypoxia and ischemia, finally leading to necrosis. A pathological evaluation revealed similar necrosis in tumors with or without a metformin treatnemt (Fig 5C), even though tumors in the case of the metformin treatment were much smaller. One of the effects of AMPK is to inhibit angiogenesis [29], and Xavier reported that metformin could inhibit angiogenesis [34]. By using an angiogenesis antibody array, Kobayashi and colleagues identified several angiogenesis related proteins that are down-regulated in metformin-treated ESCC cells[17]. Furthermore, metformin was found to induce the up-regulation of p53 and p21^CIP1^ in surrounding areas of necrosis (Fig 7C). P53 and p21^CIP1^ were reported to inhibit angiogenesis by suppressing the production of the vascular endothelial growth factor[35,36]. Based on the above findings, our data indicate that metformin might reduce angiogenesis and promote necrosis in ESCC through the up-regulation of p53 and p21^CIP1^ expression. Taken together, other than suppressing cell growth, the anti-angiogenesis effect of metformin should not be neglected.

In addition to efficacy, safety is paramount in the use of antitumor therapeutics. Metformin has generally been considered to be a safe drug during its 50-year anti-hyperglycemic history. Although the dosage of metformin (250mg/kg/d) used in this *in vivo* study was beyond the physiological level, no significant fluctuation in body weight, fasting serum glucose levels, appetite and reactions were detected in the nude mice. Our findings suggest that metformin might be effective and relatively safe for the treatment of ESCC.

Another finding is that metformin induces G0/G1 cell cycle arrest to restrict proliferation without having any adverse effect on animals. The underlying mechanisms involved AMPK activation, the up-regulation of p53, p21^CIP1^ and p27^KIP1^, as well as the down-regulation of cyclinD1. This study revealed the existence of a link between metformin, AMPK and the restriction point in ESCC, and highlights the fact that metformin might be a potential therapeutic option for the treatment of ESCC.
